# Macrophages in obesity are characterised by increased IL-1β response to calcium-sensing receptor signals

**DOI:** 10.1038/s41366-022-01135-x

**Published:** 2022-08-05

**Authors:** Stephan Thrum, Miriam Sommer, Nora Raulien, Martin Gericke, Lucas Massier, Peter Kovacs, Marco Krasselt, Kathrin Landgraf, Antje Körner, Arne Dietrich, Matthias Blüher, Manuela Rossol, Ulf Wagner

**Affiliations:** 1 Medical Department III, Leipzig University, Leipzig, Germany; 2 Integrated Research and Treatment Center (IFB) Adiposity Diseases, University of Leipzig, Leipzig, Germany; 3 Institute of Anatomy and Cell Biology, Martin-Luther-University Halle-Wittenberg, Halle, Germany; 4 Center for Pediatric Research Leipzig, University Hospital for Children & Adolescents, Department of Women’s and Child Health, University of Leipzig, Leipzig, Germany; 5 Clinic for Visceral, Transplantation and Thorax and Vascular Surgery, Leipzig University, Leipzig, Germany

**Keywords:** Obesity, Inflammatory diseases, Obesity, Preclinical research

## Abstract

**Objective:**

Obesity is complicated by inflammatory activation of the innate immune system. Stimulation of the calcium-sensing receptor (CaSR) by extra-cellular calcium ions ([Ca^2+^]_ex_) can trigger NLRP3 inflammasome activation and inflammation. We hypothesised, that this mechanism might contribute to the activation of adipose tissue (AT) in obesity, and investigated [Ca^2+^]_ex_-induced, CaSR mediated IL-1β release by macrophages in obesity.

**Methods:**

[Ca^2+^]_ex_-induced IL-1β release was investigated in monocyte-derived macrophages (MDM) generated from peripheral blood of patients with obesity and from normal-weight controls. Visceral and subcutaneous AT biosamples were stimulated with [Ca^2+^]_ex_, and IL-1β release, as well as expression of NLRP3 inflammasome and cytokine genes, was determined.

**Results:**

Both MDM and AT readily responded with concentration-dependent IL-1β release already at low, near physiological concentrations to addition of [Ca^2+^]_ex_, which was more than 80 fold higher than the LPS-induced effect. IL-1β levels induced by [Ca^2+^]_ex_ were significantly higher not only in MDM from patients with obesity compared to controls, but also in visceral versus subcutaneous AT. This fat-depot difference was also reflected by mRNA expression levels of inflammasome and cytokine genes.

**Conclusions:**

Obesity renders macrophages more susceptible to [Ca^2+^]_ex_-induced IL-1β release and pyroptosis. Increased susceptibility was independent of the response to LPS and circulating CRP arguing against mere pro-inflammatory pre-activation of monocytes. Instead, we propose that CaSR mediated signalling is relevant for the deleterious innate immune activation in obesity.

## Introduction

Severe obesity is not only characterised by mere excessive accumulation of body fat, but furthermore by accompanying immune cell infiltration in adipose tissue and obesity-related inflammation [1, 2]. The resulting low-grade systemic inflammation has been linked to clinical complications of obesity, particularly type 2 diabetes and atherosclerotic cardiovascular disease [3, 4].

Over the last years, compelling evidence has been accumulated showing the involvement of the NLR family pyrin domain containing 3 (NLRP3) inflammasome in the development of diet-induced obesity, insulin resistance and type 2 diabetes [5–7] (for review, see [8]). Evidence comes from the increased expression of NLRP3 in adipose tissue (AT) from humans and mouse models with obesity [9–13] and from the demonstrated role of NLRP3 as an important regulator of adipocyte differentiation [6]. NLRP3 activation in obesity has been described to occur in adipocytes [10, 11], but also in adipose tissue macrophages (ATM) [7].

The NLRP3 inflammasome activates Caspase 1 and causes release of IL-1β, which in turn has been identified as the major driving force behind leukocytosis in obesity due to its stimulatory effect on myeloid stem cells [14], but also as contributing factor to the development of type 2 diabetes [7, 15]. Murine studies showed that genetic deficiency for NLRP3 protects mice against high fat diet-induced obesity and insulin resistance [7, 16–18], while NLRP3 knockdown reduces AT inflammation and extracellular matrix remodelling in AT samples from people with obesity [19]. Associations between genetic polymorphisms in the NLRP3 gene and the development of insulin resistance and type 2 diabetes [20, 21] have also been described, which indicates its pathogenetic relevance.

The calcium-sensing receptor (CaSR) is a G protein-coupled receptor originally discovered in the parathyroid gland, which is responsible for monitoring and regulation of calcium concentrations in the blood. More recently, it has been found to be ubiquitously expressed in many cell types including myeloid cells and adipocytes, and to be involved in the pathogenesis of inflammatory diseases including allergic asthma [22] and inflammatory lung disease [23], myocardial infarction [24], rheumatoid arthritis [25, 26], type 2 diabetes mellitus and atherosclerosis [27].

The pro-inflammatory effects of CaSR in monocytes are mediated by activation of the NLRP3 inflammasome and subsequent IL-1β release, due to a Gα_q_-mediated transmembrane signal triggered after ligation of the receptor by extracellular calcium ions ([Ca^2+^]_ex_) [28, 29]. More recently, we could further elucidate the mechanism behind [Ca^2+^]_ex_ induced IL-1β release by showing that CaSR mediated macropinocytosis of calciprotein particles drives this inflammasome activation and subsequent cytokine release in monocytes [26].

In AT in obesity, CaSR expression has been reported to be involved in AT inflammation, since it is upregulated on adipocytes in response to inflammatory cytokines [30] and triggers their proinflammatory response by activating the NLRP3 inflammasome [31, 32]. Recently, inflammatory cytokine production in LS14 preadipocytes has also been reported to increase, if the cells are cultured in cell-cell contact with monocytic THP-1 cells pre-treated by CaSR ligation [33]. However, no data on CaSR mediated NLRP3 inflammasome activation of macrophages in obesity has been published.

The aim of the study was, therefore, to investigate the response of monocyte-derived macrophages and adipose tissue samples from people with obesity to increases in [Ca^2+^]_ex_ in order to test the possible contribution of CaSR to local and systemic inflammation in obesity. We show, that the [Ca^2+^]_ex_-induced IL-1β response of macrophages is increased in obesity compared to people who were not obese, and in visceral compared to subcutaneous AT. We propose, that [Ca^2+^]_ex_-induced, CaSR mediated NLRP3 activation of macrophages in obesity is a relevant trigger of local and systemic inflammation, which might be targeted therapeutically.

## Material and methods

### Individuals

In cooperation with the Integrated Research and Treatment Center (IFB) AdiposityDiseases of the Medical Faculty of the University Leipzig adult individuals with obesity were recruited for AT culture and for the later clinical study on macrophages reagibility. The classification of obesity was done according to the definition of the World Health Organization based on the body mass index (BMI; body weight in kilograms, divided by height in metres squared) ≥ 30 kg/m².

Exclusion criteria were pregnancy, operation during last 4 weeks, endocrinologic disorders influencing calcium homoeostasis, chronic-inflammatory diseases, known infection during the last 4 weeks, hypoproteinaemia, renal insufficiency (glomerular filtration rate ≤30 ml/min/1.73 m²), leukopenia (≤3 per µl), known malignant diseases, and drugs influencing calcium signalling/metabolism or immune system (immunosuppressive, diuretic, calcium, vitamin D, parathormone, calcium channel blocker).

The experimental design of the clinical study has been approved by the ethics committee of the University of Leipzig (AZ 044-16-ff). Informed consent was obtained from all individuals before the enrolment in the study.

### Clinical-experimental study

In a monocentric, prospective, clinical experimental study 31 people with obesity and 23 people who were not obese were recruited. The cohort of people with obesity was subdivided into an inflammatory [20] and a noninflammatory [11] group, differentiated by a C-reactive protein (CRP) ≥5 mg/dl. Cohorts were comparable in age and gender.

All individuals underwent a venous blood collection in the fasted state (empty stomach for ≥6 h, serum, EDTA-plasma und heparin-plasma), questionnaire for medical history and physical examination (including vital signs, waist- and hip-circumference, bioelectrical impedance analysis).

Blood analysis for routine parameters was performed by our hospitals laboratory (Institute for Laboratory Medicine, Clinical Chemistry and Molecular Diagnostics).

### Monocyte isolation and macrophages maturation

Following venous blood collection in EDTA-containing tubes, PBMCs were obtained by Ficoll-Paque (GE Healthcare, Chalfont St Giles, UK) density gradient centrifugation. After repeated washing in PBS containing 1mM EDTA, untouched monocytes were isolated by negative magnetic depletion using hapten-conjugated CD3, CD7, CD16, CD19, CD56, CD123 and Glycophorin A and a magnetic cell separator (Human Monocyte Isolation Kit II and MS Columns, Miltenyi Biotec, Bergisch Gladbach, Germany) according to the manufacturer’s protocol.

For maturation towards macrophages, monocytes were handled according to the description by Menck et al. [34]. In brief, about 10 million monocytes were transferred in FEP-coated cell culture bags (#32C, Saint-Gobain, La Défense, France) and seeded in cell culture media (25 ml per 10 million cells). Cell culture media contains RPMI-1640 including 2 mM L-glutamine (#R7388, Thermo Fisher Scientific, Waltham, Massachusetts, USA) with non-essential amino acids, 1 mM sodium pyruvate, mercaptoethanol 40µM (all Gibco, Thermo Fisher Scientific, Waltham, Massachusetts, USA), NaHCO_3_ 0.2 g/l (MilliporeSigma, St. Louis, Missouri, USA) and 10% autologous, heat-inactivated serum from the donor. Bags were incubated for 7 days at 37 °C with 5% CO_2_. For harvest monocyte-derived macrophages (MDM) were detached by ice incubation and minimal pressure, aspirated and washed in PBS containing EDTA again repeatedly.

### Stimulation experiments and cytokine measurement

For cytokine analysis, 80 × 10^3^ MDM per 200 µl cell culture media (RPMI-1640 containing 10% FCS) were cultured at 37 °C and 5% CO_2_ in 96-well plates at least in duplicates. Twenty hours after stimulation with 0.1–100 EU/ml lipopolysaccharide (LPS-EB Ultrapure, Invivogen, San Diego, CA, USA) and or calcium chloride, supernatants were removed, stored at −80 °C and later analysed by ELISA for IL-1β, TNF and IL-6 (BD Biosciences Pharmingen, San Diego, CA, USA) following the instructions of the assay.

Inhibition of CaSR and Caspase 1 was done by 20 µM Calhex231 (#4387, Tocris Bioscience, Bristol, UK) and 10µM Z-YVAD-FMK (sc-3071, Santa Cruz Biotechnology, Dallas, Texas, USA), respectively. The DMSO-diluted antagonists were added prior to calcium chloride. DMSO in the same dilution served as control.

### Costimulatory effects

The effects of fatty acids (FA), adipokines and hypoxia on MDM reagibility were further analysed.

Therefore the saturated FA palmitate (P9767, MilliporeSigma, St. Louis, Missouri, USA) and its unsaturated analogue palmitoleic acid (P9417, MilliporeSigma, St. Louis, Missouri, USA) were solubilized in 70% ethanol as stock solutions of 50mM. BSA (A3294, MilliporeSigma, St. Louis, Missouri, USA) was dissolved 5% in cell assay medium RPMI-1640. Stock solutions of fatty acids were added to the BSA medium to achieve a molar ratio of FA–BSA of 4:1. The pH was adjusted to 7.4 with 0.25 M NaOH and the solution was filtered using a 0.2 µm low protein-binding filter (Filtropur S 0.2, Sarstedt, Nümbrecht, Germany). BSA medium alone was used in control solutions.

The four adipokines Leptin, Chemerin (both R&D Systems, Minneapolis, Minnesota, USA), FABP4 and Progranulin (both Adipogen Corporation, San Diego, CA, USA) have been diluted in TRIS (Leptin, FABP4) and PBS (Chemerin and Progranulin) respectively. Experiments were done using named adipokines as costimulatory substance during stimulation period or during maturation of monocytes towards macrophages.

In a separate experiment hypoxic conditions were maintained by transfer of cell culture plates into bags, aspiration of room air and insufflation of hypoxic air (5% CO_2_, 1% O_2_, 94% N_2_).

### Co-culture MDM with adipocytes

For co-culture experiments adipocytes were differentiated from the Simpson–Golabi–Behmel syndrome (SGBS) preadipocyte cell line [35]. Cells were cultured in basal SGBS medium consisting of DMEM/Ham F12 medium (Thermo Fisher Scientific, Waltham, Massachusetts, USA) supplemented with 33 µM biotin and 17 µM pantothenic acid. Cells were differentiated into adipocytes as previously described [36]. Briefly, SGBS preadipocytes were grown to confluence in basal medium supplemented with 10% FCS. Adipocyte differentiation was induced under serum-free conditions by supplementing basal medium with 20 nM insulin, 0.2 nM triiodothyronine, 100 nM hydrocortisone, and 0.13 nM apo-transferrin. For the first 4 days of differentiation, 2 µM rosiglitazone, 25 nM dexamethasone and 500 µM 3-isobutyl-1-methylxanthine were additionally added.

25 × 10^3^ MDM per 2 ml cell culture media (RPMI-1640 containing 10% FCS) were cultured on 50 × 10³ SBGS-cells seeded in 12-well plates and incubated 37 °C and 5% CO_2_ for 24 h, followed by a 20 h stimulation as described above.

### Adipose tissue culture

Visceral (right upper greater omentum) and subcutaneous AT was obtained from people with obesity, who underwent elective bariatric surgery at University Hospital Leipzig. Fat pads were transported sterile in RPMI 1640 alone. After washing in PBS, about 100mg in weight fat pieces were separated from the pad, further minced into 2–3 mm³ fragments, and incubated at 2ml culture media (RPMI 1640 supplemented with 10% FCS), 37 °C and 5% CO_2_ for 24 h at a 12-well culture plate. Hereafter medium was refreshed by 10 µl culture media per mg tissue and was stimulated 20 h using 100 EU/ml lipopolysaccharide and/or 1.5 mM calcium chloride respectively. Finally, supernatants were removed, stored at −80 °C and later analysed by ELISA for IL-1β, TNF and IL-6 (BD Biosciences Pharmingen, San Diego, CA, USA) following the instructions of the assay.

### Gene expression analysis

AT biopsies (subcutaneous and omental-visceral) were obtained from 71 adult individuals undergoing bariatric surgery at the University Hospital Leipzig. RNA was isolated using the RNeasy Lipid Tissue Mini Kit (Qiagen, Hilden, Germany) according to the manufacturers’ protocol and reversely transcribed with Super Script III Reverse Transcriptase (Thermo Fisher Scientific, Waltham, Massachusetts, USA). Gene expression experiments were run on a LightCycler 480 (F. Hoffmann-La Roche, Basel, Switzerland) with TaqMan Assays (NLRP3: Hs00918082_m1; PYCARD: Hs01547324_gH; CASP1: Hs00354836_m1; IL-1β: Hs01555410_m1; IL-6: Hs00174131_m1; TNF: Hs00174128_m1; Thermo Fisher Scientific, Waltham, Massachusetts, USA). GAPDH (Hs02786624_g1) was chosen as reference gene, after testing the stability of nine reference genes in a sub cohort. Expression data were analysed with the ΔΔCt method according to Pfaffl [37]. All individuals gave written informed consent before taking part in this study.

### Immunhistochemistry

Sample aliquots were incubated in 4% paraformaldehyde in PBS for 24 h and embedded in paraffin. 6 µm slides were used and treated with DAKO retrieval solution (pH = 9, Agilent, Santa Clara, CA, USA) for 30 min by applying hot steam. Adipocytes were stained with anti-perilipin-1 (goat, 1:200, Abcam, Cambridge, UK, #ab61682) and macrophages with anti-Iba1 (rabbit, 1:500, WAKO, #019-19741) at 4 °C overnight. Supervised automated analysis was performed using CellSens software (OLYMPUS Life Science, Shinjuku, Japan) to assess adipocyte count and diameter as well as counts of macrophages (*n* = 60).

### Statistical analysis

For statistical analysis, Prism 5 (GraphPad Software Inc., USA) was used. Gaussian distribution was verified using D’Agostino-Pearson normality test.

In case of normal distribution Student’s paired or unpaired *t*-test was used. Gene expression between subcutaneous and visceral tissue was compared with Wilcoxon signed-rank test. Otherwise, a Mann–Whitney *U* test was performed. A *p* value less than 0.05 was considered statistically significant. Subject number was estimated by power analysis (effect size 0.5, alpha level 0.05, power 0.8).

## Results

### Obesity increases the [Ca^2+^]_ex_-induced IL-1ß release of MDM

**Fig. 1:**
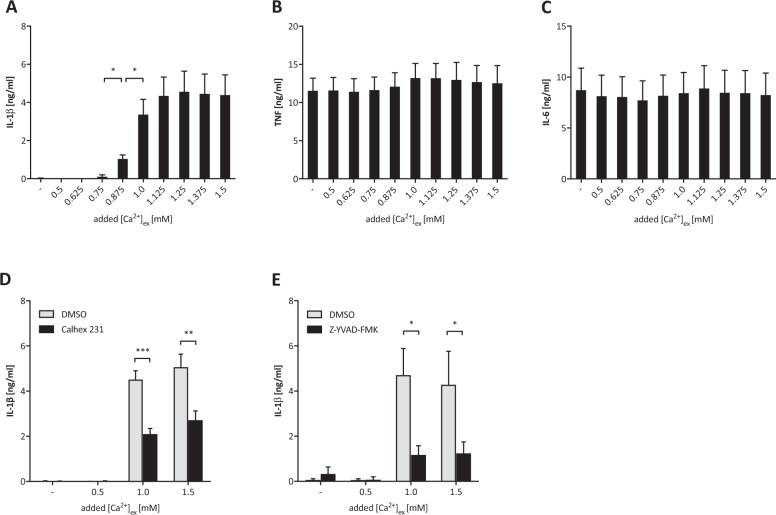
Stimulation of monocyte-derived macrophages with increasing [Ca^2+^]_ex_ induces calcium-sensing receptor (CaSR)-dependent IL-1β release and Caspase-1 activation. Monocyte-derived macrophages (MDM) generated from peripheral blood monocytes from healthy donors over 7 days were stimulated with LPS and additional [Ca^2+^]_ex_ as indicated. Concentrations of IL-1β (**A**), TNF (**B**) and IL-6 (**C**) were determined in the supernatant after 20 h (*n* = 6). Influence of the CaSR inhibitor Calhex 231 (20 µM, **D**) and the Caspase-1 inhibitor Z-YVAD-FMK (10 µM, **E**) on calcium-induced IL-1β release (*n* = 3). Levels of significance as indicated (*p < 0.05, **p < 0.01, ***p < 0.001), experiments in **D** and **E** were done in triplicates.

We and others have shown, that LPS primed monocytes, as well as murine macrophages, respond to increased [Ca^2+^]_ex_ by activating the NLRP3 inflammasome and release of IL-1ß [28, 29]. Here, we report that human MDM respond similarly to increased [Ca^2+^]_ex_ with IL-1β release. Stimulation started at 0.8 mM and peaked at 1.1 mM [Ca^2+^] (Fig. 1A, *n* = 6), which is earlier than in monocytes [28, 29], and occurred already at extremely low LPS concentrations of 0.1 EU per mL (Supplementary Fig. 1). No effect of [Ca^2+^]_ex_ on the LPS-induced TNF and IL-6 secretion was seen (Fig. 1B, C, Supplementary Fig. 1, *n* = 6). IL-1β release was CaSR-mediated and inflammasome-dependent, since Calhex 231, a specific negative allosteric modulator of the CaSR, and the Caspase-1 inhibitor Z-YVAD-FMK both abrogated the effect (Fig. 1D, E, *n* = 3).

To investigate the [Ca^2+^]_ex_-induced IL-1β response in obesity, macrophages were generated from monocytes from healthy individuals who did not have obesity and from people with obesity in vitro in the presence of autologous serum in order to recapitulate the metabolic conditions in vivo in the blood and in AT (Supplementary Fig. 2). To provide an opportunity to analyse the influence of systemic inflammation on the [Ca^2+^]_ex_-induced IL-1β response, patients with increased CRP values in the absence of other inflammatory conditions were preferentially recruited and constituted more than 60% of the final study population (for clinical parameters, see Table 1).

**Table 1:** Clinical characteristics of study population.

	Obese		Non-obese
	Inflamm	Non-inflamm.	
No. (Sex, F/M)	20 (17/3)	11 (11/0)	23 (20/3)
Age, y	42.3 ± 15.9	44.4 ± 14.9	40.2 ± 14.4
Body mass index (BMI), kg/m^2^	44.7 ± 6.4	40.7 ± 5.0	22.7 ± 3.0
Waist-hip ratio (WHR)	0.9 ± 0.1	0.9 ± 0.1	0.8 ± 0.1
Type 2 diabetes	5 (25.0)	3 (27.3)	0 (0.0)
Impaired glucose tolerance	4 (20.0)	1 (9.1)	0 (0.0)
CRP, mg/L	12.5 ± 5.5	2.8 ± 1.3	1.1 ± 0.5

All values are expressed as mean ± SEM and percentage (brackets). Statistics were performed as described in ‘Materials and Methods’.

**Fig. 2:**
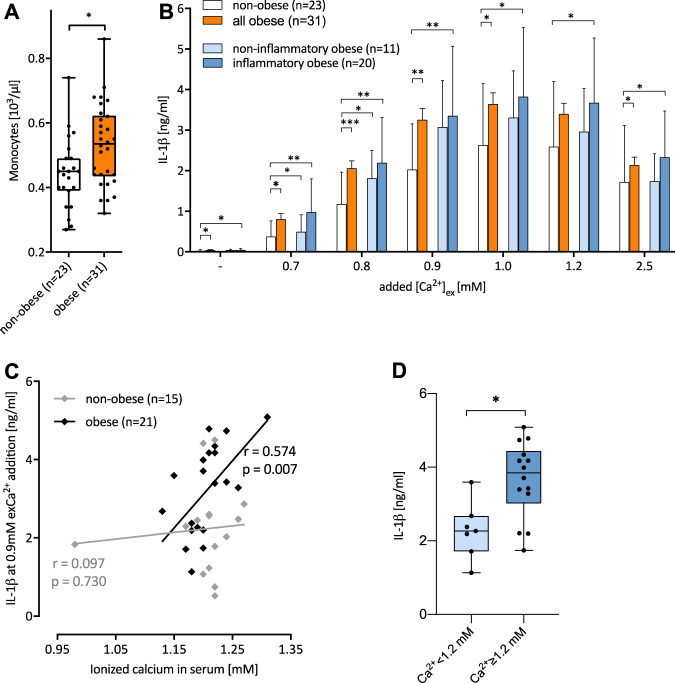
[Ca^2+^]_ex_-induced IL-1β response of MDM is increased in obesity. **A** Box plot shows absolute numbers of circulating monocytes in individuals who did not have obesity and in people with obesity as determined by differential blood count. Number of individuals and level of significance determined by Mann-Whitney *U* test as indicated. **B** Bar charts depict median and SEM of [Ca2+]ex-induced IL-1ß response of MDM generated from obese people with or without increased CRP value (inflammatory and non-inflammatory obese) and from individuals who did not have obesity. Levels of significance: *p < 0.05, **p < 0.01, determined by student’s paired *t*-test. Correlation between plasma levels of ionised Ca2+ and Ca2+-induced IL-1ß release in cohort of people with and without obesity (**C**) and Ca^2+^-induced IL-1ß release in patients (*n* = 21, 47.6% non-inflammatory and 52.6% inflammatory obesity) with ionised Ca^2+^ below and above 1.2 mM (**D**). Spearman’s rank correlation (**C**) and Mann–Whitney U test (**D**) were used.

The study confirmed higher absolute frequencies of monocytes, lymphocytes, immature granulocytes and neutrophils in the peripheral blood of people with obesity in comparison with people who were not obese as reported previously [38–42], while no differences in eosinophils and basophils were detectable (Fig. 2A and Suppl. Table 1), indicating a more proinflammatory environment in bone marrow. Other significant differences in the laboratory findings determined included increased values for glycosylated HbA1c (5.8 vs. 5.2%, *p* = 0.002) and triglycerides (1.71 vs. 1.12 mmol/l, *p* < 0.001) and decreased serum levels of Vitamin D3 (31 vs. 48 nmol/l, *p* < 0.001, Suppl. Table 2).

When [Ca^2+^]_ex_-induced IL-1ß release of MDM from people with obesity was compared to that of individuals who did not have obesity, significantly higher concentrations were found in the group with obesity for all calcium concentrations tested (Fig. 2B, *n* = 23 and 31, resp.). To investigate the role of a systemic inflammatory constellation in [Ca^2+^]_ex_-induced IL-1β release, people with inflammatory obesity (CRP values >5 mg/L) were compared to individuals with non-inflammatory obesity. Importantly, [Ca^2+^]_ex_-induced IL-1β release of MDM from non-inflammatory people with obesity did not differ from those with increased CRP values, and was still significantly increased compared to controls (Fig. 2B, *n* = 11 and 20, resp.).

No differences in plasma [Ca^2+^] concentrations were detectable between the group with and without obesity, or between people with and without elevated CRP levels (data not shown). However, [Ca^2+^]-induced IL-1ß production of MDM was found to correlate with plasma [Ca^2+^] concentrations within the cohort of obese people, but not in individuals who did not have obesity (Fig. 2C). People with a plasma [Ca^2+^] of 1.2 mM (the median of the study population) or higher were found to release significantly more [Ca^2+^]_ex_-induced IL-1β compared to those with lower [Ca^2+^] values (Fig. 2D). Monocytes for MDM might be preconditioned in vivo due to the calcium exposition or other intrinsic factors in obesity.

### [Ca^2+^]_ex_-induced IL-1β release is not influenced by cell–cell contact with adipocytes or adipose tissue factors present in obesity

**Fig. 3:**
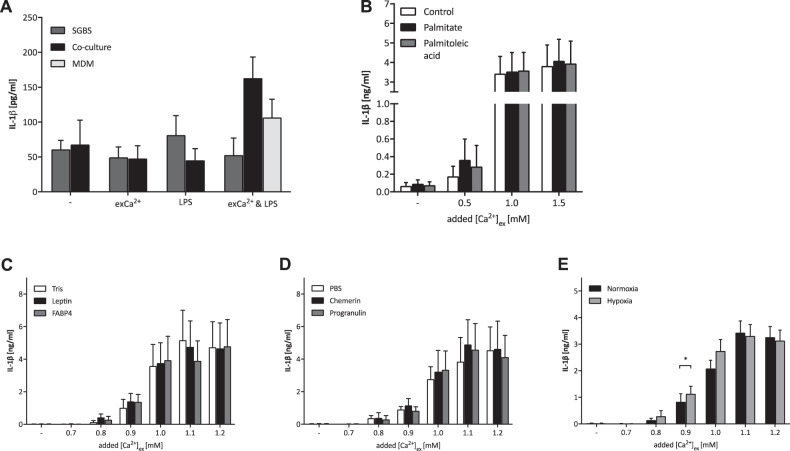
The response of MDM towards exCa^2+^ in vitro is not influenced by cell contact with adipocytes or metabolic parameters associated with adipose tissue in obesity. **A** Simpson–Golabi–Behmel syndrome (SGBS) preadipocyte cells and MDM were incubated alone or in co-culture as indicated in the legend in medium only (-) or in the presence of 1.5 mM [Ca^2+^]ex, 100 EU/ml LPS, or both (*n* = 4). [Ca^2+^]ex-induced IL-1ß response of MDM stimulated in the absence of presence of the indicated fatty acids (**B**, *n* = 4), adipokines (**C**, **D**, *n* = 4) or hypoxia (**E**, *n* = 8). [Ca^2+^]ex was added as indicated on the *x*-axis. Bar charts depict mean and SEM, level of significance as indicated (*p < 0.05).

To investigate the influence of macrophage–adipocyte cell-cell-contact on [Ca^2+^]_ex_-induced IL-1β release, a co-culture system of the human white adipocyte cell line SGBS with monocyte-derived human macrophages from healthy individuals was established. When cultured alone, differentiated SGBS adipocytes produced low amounts of IL-1β in response to LPS (Fig. 3A). Increased [Ca^2+^]_ex_ triggered IL-1β release in the co-cultures, while it had no influence on SGBS cells incubated alone.

Local metabolic conditions in AT in obesity are characterised by increased glucose availability, but also by increased concentrations of lipids and fatty acids. Among the latter, palmitate is best characterised for a pro-inflammatory, stimulatory effect on macrophages, and was therefore tested for its influence on [Ca^2+^]_ex_-induced IL-1β release of MDM [43]. The saturated fatty acid palmitate had no influence on the [Ca^2+^]_ex_-induced IL-1β release of MDM, and neither had unsaturated palmitoleic acid (Fig. 3B, *n* = 4). Furthermore, when MDM were generated in the presence of palmitate throughout maturation, no effect on [Ca^2+^]_ex_-induced IL-1β release was discernible (data not shown).

Distinct adipokines are also known for their profound effect on endocrine and immunological pathways in obesity. We therefore investigated the influence of leptin, FABP4, chemerin and progranulin on the [Ca^2+^]_ex_-induced IL-1β response of MDM. Interestingly, neither adipokine affected [Ca^2+^]_ex_-induced IL-1β release of macrophages (Fig. 3C, D, *n* = 4). Again, when MDM was generated in the presence of adipokines throughout the period of maturation—in order to simulate the in vivo conditions in obesity—the resulting macrophages were also not different in their response towards increased [Ca^2+^]_ex_ (Suppl. Fig. 5).

The local environment in AT is known not only for an excess nutrient supply, but also for localised hypoxic conditions [44], which are thought to influence functional parameters. Analysis of [Ca^2+^]_ex_-induced IL-1β release under conditions of hypoxia showed indeed increased IL-1β concentrations at certain concentrations (Fig. 3E, *n* = 8).

### [Ca^2+^]_ex_-induced IL-1β production and inflammasome gene expression of adipose tissue in obesity

Based on the finding of an increased exCa^2+^-induced IL-1ß response of macrophages derived from peripheral blood monocyte from individuals with obesity, we investigated the [Ca^2+^]_ex_-induced IL-1β release in subcutaneous and visceral AT samples obtained from patients undergoing bariatric surgery.

**Fig. 4:**
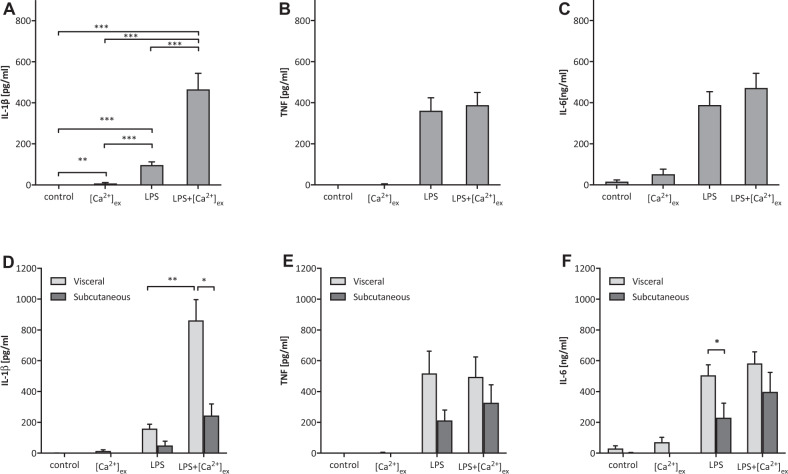
[Ca^2+^]ex-induced response of visceral and subcutaneous adipose tissue specimens. Adipose tissue samples from the omentum majus (*n* = 6) and from subcutaneous locations (*n* = 14) were cultured in vitro as described in material and methods, and stimulated with LPS with or without added [Ca^2+^]ex (2.5 mM to the culture media, *n* = 20). **D**–**F** Results are analysed separately for subcutaneous and visceral specimens (*n* = 5). Bar charts depict mean and SEM of IL-1β (**A**, **D**), TNF (**B**, **E**) and IL-6 (**C**, **F**) concentrations determined after 16 h in culture. Level of significance: *p < 0.05, **p < 0.01, **p < 0.01, ***p < 0.001).

Stimulation with LPS and [Ca^2+^]_ex_ induced a significantly higher IL-1ß response in AT samples compared to LPS alone (Fig. 4A). The cytokines TNF and IL-6 were found to be produced already after stimulation with LPS alone, but [Ca^2+^]_ex_-induced increase was not detectable (Fig. 4B, C, *n* = 20).

When visceral and subcutaneous tissue sample were analysed separately, a significantly higher [Ca^2+^]_ex_-induced IL-1β release was detectable in visceral AT samples than in subcutaneous AT from the same people with obesity (Fig. 4D). No significant differences in [Ca^2+^]_ex_-induced TNF and IL-6 secretion between visceral and subcutaneous AT were discernible (Fig. 4E, F, *n* = 5).

**Fig. 5:**
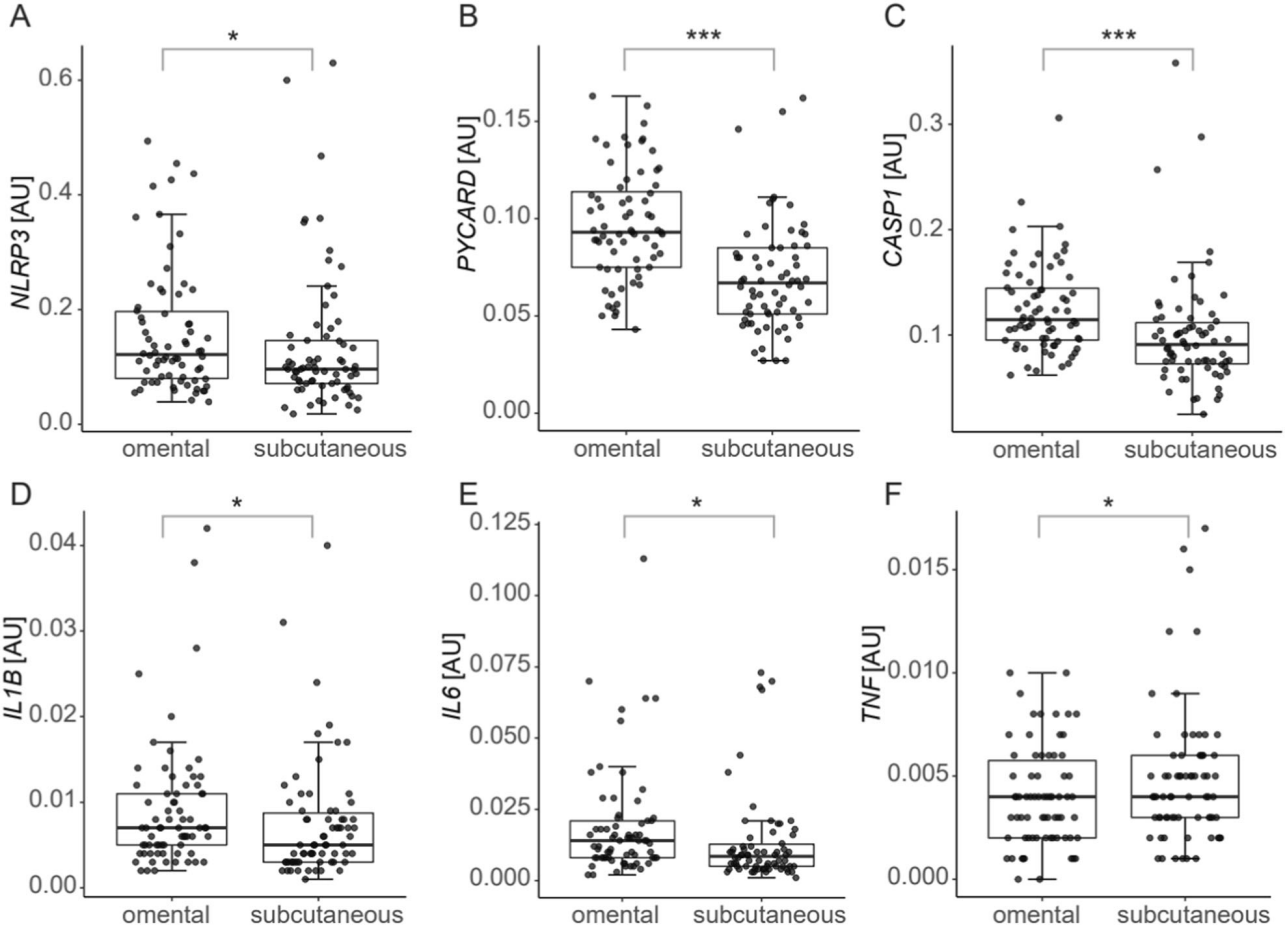
Gene expression analysis in adipose tissue samples obtained during bariatric surgery from the omentum majus (visceral) and from subcutaneous adipose tissue. Box plots depict mean and interquartile range of RNA expression of the genes indicated. Level of significance as indicated (*p < 0.05, **p < 0.01, ***p < 0.001, *n* = 71).

In order to elucidate the mechanism behind the increased pro-inflammatory [Ca^2+^]_ex_-induced IL-1β release in visceral AT in obesity, RNA expression was analysed by realtime PCR in the tissue samples. The results showed, that mRNA expression of the inflammasome components NLRP3, PYCARD (the gene coding for ASC, Apoptosis-associated speck-like protein containing a CARD) and Caspase 1 as well as of the Caspase 1-dependent cytokine IL-1β were all significantly higher in visceral compared to subcutaneous AT (Fig. 5, *n* = 71). In addition, the proinflammatory Caspase 1-independent cytokines TNF and IL-6 were also significantly increased in visceral AT.

The frequency of macrophages in AT has an obvious influence on mRNA copy numbers of the pro-inflammatory genes investigated, and has been reported to be increased in visceral AT, probably as a result from immigration of peripheral blood monocytes into the tissue [44–46]. In order to explore the influence of macrophage numbers in the tissue on pro-inflammatory gene expression, ATMs were quantified by immunohistochemistry. Indeed, visceral AT contained higher macrophage numbers per 100 adipocytes compared to subcutaneous AT (median 37.5, interquartile range IQR 22.0–51.79; vs. median 21.6, IQR 13.4–39.5; *p* = 0.012 by paired Wilcoxon test, *n* = 60, see Suppl. Fig. 3). Furthermore, correlation analysis showed gene expression of NLRP3, PYCARD and IL-1B to correlate with the frequency of macrophages in subcutaneous AT (*r* = 0.286, *p* = 0.024; *r* = 0.348, *p* = 0.006; and *r* = 0.398, *p* = 0.001, respectively), but no such correlation was detectable for visceral AT (Suppl. Fig. 3). Accordingly, increased macrophage numbers might contribute to the observed significant differences in gene expression and [Ca^2+^]_ex_-induced IL-1β release, but are unlikely to be the sole explanation [47].

## Discussion

Stimulation with increased concentrations of extracellular Ca^2+^ ions has been reported to be a strong pro-inflammatory signal for monocytes, but also for adipocyte cell lines [32], which triggers NLRP3 inflammasome assembly and IL-1β release. Recently, we could further elucidate the underlying mechanism in monocytes by showing, that formation of calciprotein particles in the presence of phosphate is required for this stimulation, and that an [Ca^2+^]_ex_-induced CaSR signal triggers macropinocytosis of these particles [26]. We report here, that macrophages in obesity also respond with [Ca^2+^]_ex_-induced IL-1β release. This stimulation occurs in the cohort of people with obesity already at lower [Ca^2+^]_ex_ than in individuals who did not have obesity, and is stronger at equal [Ca^2+^]_ex_ concentrations in macrophages from people with obesity than in controls. This effect is mediated by [Ca^2+^]_ex_-induced signalling of the CaSR, since it can be inhibited by the pharmacologic antagonist of the receptor, Calhex 231.

The observation, that the increased ex vivo response towards [Ca^2+^]_ex_ was more prominent in visceral than in subcutaneous AT—due to either increased macrophage numbers or intrinsic hyper-reagibility—emphasises the potential relevance of this response, since visceral AT is the site of the highest pro-inflammatory activity in obesity. There, ATM produces MCP-1 and CSF-1 and accumulates both due to monocyte immigration from peripheral blood and proliferation of resident macrophages [48], which has specifically been brought into context of pro-inflammatory and atherosclerotic comorbidities of obesity.

In addition, the different response of visceral versus subcutaneous AT suggests that not systemic calcium concentrations as determined in the serum of peripheral blood are most relevant for [Ca^2+^]_ex_-induced IL-1β release in obesity, but that local tissue concentrations could be the trigger. Mechanistically, increased [Ca^2+^]_ex_ in the vicinity of activated or dying cells, possibly due to chronic inflammation, in AT in obesity could contribute to increased CaSR signalling [49].

Although people with obesity as a cohort did not differ from individuals who did not have obesity in their plasma concentrations of ionised calcium, we still found a close correlation of plasma calcium levels with the [Ca^2+^]_ex_-induced IL-1β release of MDM from the group with obesity. One possibility to interpret this finding is, that equal concentrations of [Ca^2+^]_ex_ induce more IL-1β release in obesity than in controls. If such an increased propensity to react to [Ca^2+^]_ex_ is characteristic for monocytes and MDM in obesity, then even physiologic levels of [Ca^2+^]_ex_ in tissue might be sufficient to trigger an inflammatory response. Possible explanations for such an increased [Ca^2+^]_ex_-induced response are altered signalling or up-regulated expression of the CaSR. The observation that monocytes and MDM from patients with obesity show an increased [Ca^2+^]_ex_-induced IL-1β response already before they enter the tissue indicates, that those alteration occur during myelopoiesis in the bone marrow. The observed monocytosis in obesity suggests increased and possibly altered myelopoiesis, perhaps due to a more proinflammatory environment in bone marrow in obesity.

Alternatively, it is also feasible, that not calcium ions but other agonists at the CaSR, such as eosinophil cationic proteins, spermine and polyamine derivatives produced by inflammatory effector cells could be triggering NLRP3 inflammasome activation and IL-1β release in vivo.

There have been reports of increased CaSR expression on monocytes from people suffering from type 2 diabetes, atherosclerosis, and rheumatoid arthritis [25, 27]. Recently, we could show that in rheumatoid arthritis, the increased [Ca^2+^]_ex_-induced IL-1β release of monocytes is caused, in part, by increased CaSR expression. Similarly, CaSR over-expression on peripheral blood monocytes or MDM in obesity could lead to increased G protein-coupled receptor (GPCR) signalling and explain the observed increase of [Ca^2+^]_ex_-induced IL-1β release. The increased [Ca^2+^]_ex_-induced IL-1β release of visceral compared to subcutaneous AT samples could in part be due to increased macrophage numbers, which was not investigated in detail in this study without ATM separation. But upregulated expression of inflammasome components and potentially also of the CaSR in the tissue might also be contributing to inflammasome activation already at lower [Ca^2+^]_ex_ concentrations. In this scenario, physiological [Ca^2+^]_ex_ levels as they are assumed to be present in tissue [48] would be sufficient to induce NLRP3 inflammasome activation, and the observed stimulatory effect in vitro already at concentrations as low as 0.8 mM supports this hypothesis.

Inflammasome activation and IL-1β release always require a ‘first signal’ provided by pathogen-associated or danger-associated molecular patterns, which in vitro is usually provided by priming with LPS. While monocytes were previously found to require at least LPS concentrations of 100 EU per mL for [Ca^2+^]_ex_-induced NLRP3 inflammasome activation [26], we show here that the response of macrophages in obesity occurs already in a very low LPS concentration range which is comparable to what has been reported for postprandial LPS levels in the serum of people with obesity and diabetes [50]. In view of the recently revealed presence of bacteria and bacterial DNA in AT in obesity [51, 52], it therefore appears feasible that the required ‘first signal’ for NLRP3 inflammasome activation is provided to ATM in obesity. Data presented by caspase-inhibition and gene expression analyses here indicate involvement of NLRP3, but a contribution of other inflammasomes can not be exclude conclusively.

No correlation was detectable between clinical parameters of obesity like BMI or waist circumference and the magnitude of [Ca^2+^]_ex_-induced IL-1ß release, indicating that the increased [Ca^2+^]_ex_-induced response of MDM and AT is a feature of severe obesity per se, which is not quantitatively linked to excess body weight, and which might therefore already be present at early stages of the disease. Accordingly, [Ca^2+^]_ex_-induced IL-1ß release could be useful as a diagnostic parameter, by identifying people at risk for obesity-related co-morbidities requiring timely diagnostic tests and possibly therapeutic intervention.

Recruitment of the study population was deliberately skewed towards a more inflammatory phenotype of the disease, due to the presumed associations between NLRP3 activation, IL-1ß release and co-morbidities. Our subgroup analysis clearly indicated, however, that increased [Ca^2+^]_ex_-induced, CaSR mediated IL-1ß release was not limited to people with a clinically detectable inflammatory state, but was also associated with obesity in individuals with normal CRP levels. This indicates that this increased responsiveness is indeed a feature of obesity per se and not a secondary phenomenon due to the low-grade systemic metaflammation associated with this disease.

In summary, we have shown that macrophages from people with obesity, and whole tissue cultures from visceral AT, show increased [Ca^2+^]ex-induced, CaSR-mediated NLRP3 inflammasome activation and IL-1ß release when compared to people who were not obese. Accordingly, the NLRP3 inflammasome pathway as well as the resulting production of the master cytokine IL-1ß might contribute to clinically relevant consequences of inflammation in obesity.

## Supplementary information

Supplementals

## Data Availability

Requests for further information or for resources and reagents should be directed to the corresponding author.
