# Supplementary material for: Macrophages in obesity are characterised by increased IL-1β response to calcium-sensing receptor signals
Source: Int J Obes (Lond). 2022 Aug 5;46(10):1883–91. doi: 10.1038/s41366-022-01135-x (PMC9492543; doi:10.1038/s41366-022-01135-x)
Supplement: Supplementary file 1 — Supplementals [file 41366_2022_1135_MOESM1_ESM.docx]

**Supplementary figure 1**

**Supplementary figure 1: Influence of increasing LPS concentrations on [Ca^2+^]_ex_-induced IL-1β release of monocyte-derived macrophages**

1A-C: MDM generated from peripheral blood monocytes from healthy donors over 7 days were stimulated with additional [Ca^2+^]_ex_ and increasing concentrations of LPS as indicated. Concentrations of IL-1β, TNF and IL-6 were determined in the supernatant after 20 hours (n=6).

**Supplementary figure 2**


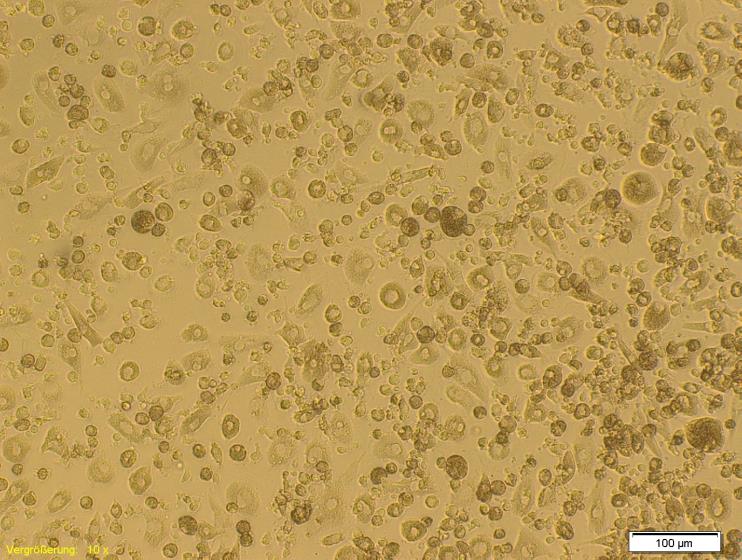

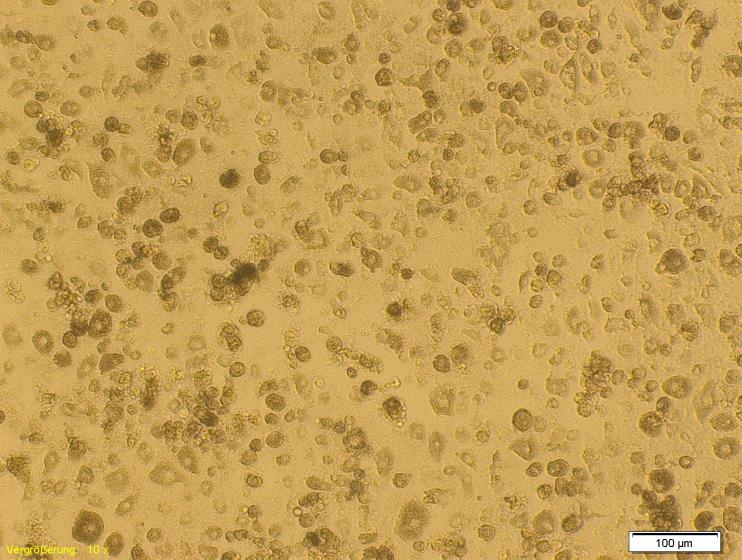

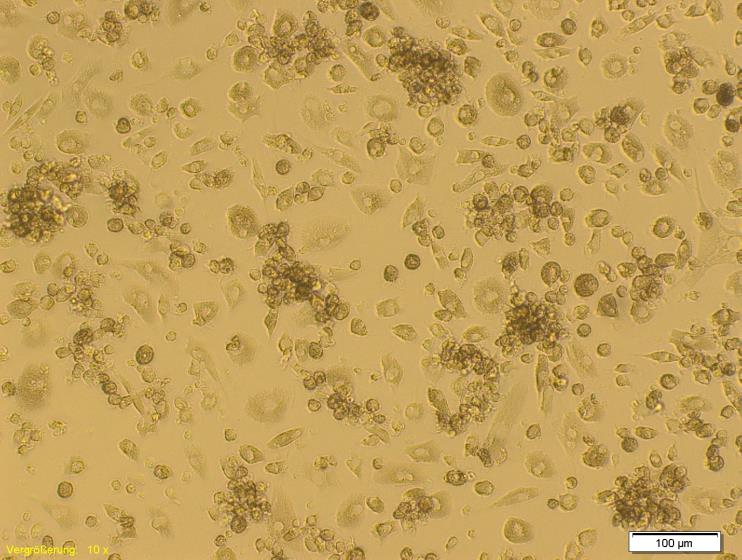

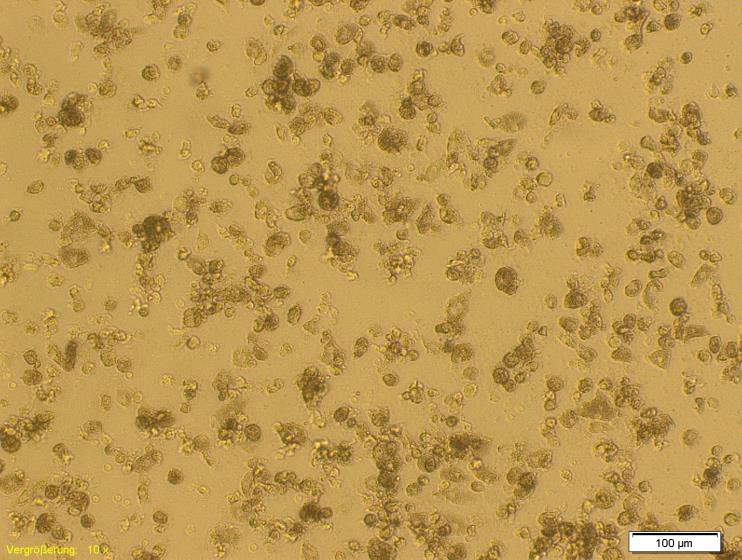


A

C

D

B

**Supplementary figure 2:** Representative images of MDM after 7d of differentiation and 20h of stimulation under tissue culture conditions described in material and methods. MDM were treated with cell culture media alone (A), exCa^2+^ (B), LPS alone (C) or with LPS and exCa^2+^ (D).

**Supplementary figure 3:**


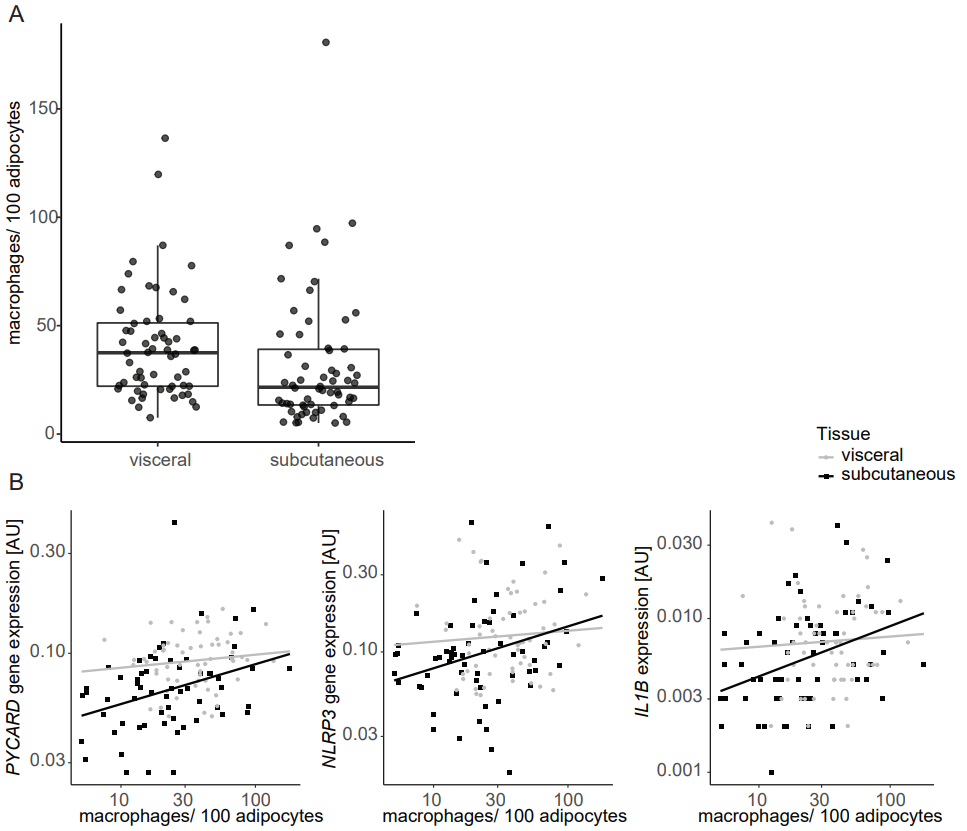


Supplementary figure 3: Frequency of macrophages in AT dependent on type of AT (A) and its influence on mRNA expression of the pro-inflammatory genes (B) (n=60).

**Supplementary figure 4:**

Supplementary figure 4: MDM generated from peripheral blood monocytes from healthy donors over 7 days were stimulated with additional 2,5 mM [Ca2+]ex and 100 EU/ml LPS. Concentrations of IL-1β and LDH-dependent cytotoxicity were measured at different time points during the course of stimulation.

**Supplementary figure 5:**

**Supplementary figure 5:** The response of MDM towards exCa^2+^ in vitro is not influenced by maturation in different adipokines. Monocyte-derived macrophages (MDM) were differentiated in the absence or presence of leptin and chemerin, respectively. Subsequent Stimulation was performed by addition of [Ca^2+^]_ex_ and 100 EU/ml LPS (n=6).

| **Supplementary table 1: Frequencies of circulating leucocytes classes** | | | |
| --- | --- | --- | --- |
|  | P value | Mean [10^9^/l]  Obese Non-Obese | |
| WBC | < 0.0001 | 8.28 | 5.65 |
| Monocytes | 0.0073 | 0.55 | 0.44 |
| Lymphocytes | < 0.0001 | 2.51 | 1.83 |
| Neutrophiles | < 0.0001 | 5.01 | 3.10 |
| Eosinophiles | 0.7009 | 0.18 | 0.20 |
| Basophiles | 0,5618 | 0.05 | 0.05 |
| Healthy individuals who did not have obesity (n = 17) and people with obesity (n = 25). Absolute numbers of circulating monocytes were determined by differential blood count. Level of significance as indicated. | | | |

| **Supplementary table 2:** **Selected laboratory results** | | | |
| --- | --- | --- | --- |
|  | P value | Mean [10^9^/l]  Obese Non-Obese | |
| HbA1c [%] | 0.002 | 5.8 | 5.2 |
| Vitamin D3 [nM] | < 0.001 | 31 | 48 |
| CRP [mg/l] | < 0.001 | 9.51 | 1.56 |
| Triglycerides [mM] | < 0.001 | 1.71 | 1.12 |
| HDL cholesterol [mM] | < 0.001 | 1,30 | 1,68 |
| LDL cholesterol [mM] | 0.255 | 3,65 | 3,29 |
| Albumin [g/l] | 0.143 | 42.8 | 44.7 |
| Healthy individuals who did not have obesity (n = 17) and people with obesity (n = 25). Level of significance as indicated. | | | |
